# Blocking ***α***V***β***3 Integrin Ligand Occupancy Inhibits the Progression of Albuminuria in Diabetic Rats

**DOI:** 10.1155/2014/421827

**Published:** 2014-10-20

**Authors:** Laura A. Maile, Katherine Gollahon, Christine Wai, Paul Dunbar, Walker Busby, David Clemmons

**Affiliations:** ^1^Department of Medicine, UNC School of Medicine, Chapel Hill, NC 27599, USA; ^2^Vascular Pharmaceuticals, Inc., 510 Meadowmont Village Circle, Suite 283, Chapel Hill, NC 27517, USA

## Abstract

This study determined if blocking ligand occupancy of the *α*V*β*3 integrin could inhibit the pathophysiologic changes that occur in the early stages of diabetic nephropathy (DN). Diabetic rats were treated with either vehicle or a monoclonal antibody that binds the *β*3 subunit of the *α*V*β*3 integrin. After 4 weeks of diabetes the urinary albumin to creatinine ratio (UACR) increased in both diabetic animals that subsequently received vehicle and in the animals that subsequently received the anti-*β*3 antibody compared with control nondiabetic rats. After 8 weeks of treatment the UACR continued to rise in the vehicle-treated rats; however it returned to levels comparable to control nondiabetic rats in rats treated with the anti-*β*3 antibody. Treatment with the antibody prevented the increase of several profibrotic proteins that have been implicated in the development of DN. Diabetes was associated with an increase in phosphorylation of the *β*3 subunit in kidney homogenates from diabetic animals, but this was prevented by the antibody treatment. This study demonstrates that, when administered after establishment of early pathophysiologic changes in renal function, the anti-*β*3 antibody reversed the effects of diabetes normalizing albuminuria and profibrotic proteins in the kidney to the levels observed in nondiabetic control animals.

## 1. Introduction

The development of new therapeutic approaches to treat diabetic nephropathy (DN) has been complicated by the multiplicity of pathophysiologic changes occurring in this disorder [1]. Furthermore, the definition of the specific cell types whose function is altered by the presence of chronic hyperglycemia and/or deficient insulin action and the determination of how abnormalities among these different cell types are coordinately regulated leading to loss of kidney function are not well defined. Among the pathophysiologic changes, activation of the polyol pathway, oxidative stress, protein kinase C, and cellular responses to glucose-induced glycated proteins have received the greatest interest [1, 2]. However, other processes such as activation of transforming growth factor-beta (TGF-*β*) leading to fibrosis and epithelial to mesenchymal transition, alterations in podocyte detachment, or loss of endothelial glycocalyx and the role of excessive accumulation of mesangial extracellular matrix are also areas of intensive investigation [2, 3]. Several types of interventions have been developed that target one or more of these pathophysiologic events. Some of these approaches reduce proteinuria, but approaches that successfully alter the long-term progression of histologic changes and more importantly achieve sustained attenuation in the rate of loss of glomerular filtration rate (GFR) have not been reported [4, 5].

The role of endothelial dysfunction in mediating the changes that occur in early DN has been intensively investigated [6]. In response to chronic hyperglycemia the glomerular endothelium undergoes multiple changes including activation of oxidative stress mechanisms, the recruitment of inflammatory cells, the generation of inflammatory cytokines, dysfunctional regulation of endothelial nitric oxide activation, and increased expression of TGF-*β* [6, 7]. These changes lead to loss of endothelial fenestrations and alterations in intercellular communication between endothelium and podocytes [8, 9]. As a result of these changes barrier function is disrupted and microalbuminuria ensues.

Although it is acknowledged that endothelial contact with the basement membrane is critical for normal endothelial and podocyte function, the role of integrin receptors which mediate cell attachment to basement membrane proteins and their role in the pathophysiologic changes that occur in the endothelium, podocytes, and mesangium in response to hyperglycemia are not well defined [10–12]. One integrin, *α*V*β*3, is expressed abundantly on endothelial cells including glomerular endothelium [13]. This integrin binds to osteopontin and thrombospondin (TS-1) whose concentrations are increased in kidneys of diabetic animals [14, 15]. Ligand occupancy of this integrin leads to changes in intracellular signaling mechanisms which predispose to significant alterations in endothelial function including changes in endothelial permeability [16]. Recently our laboratory demonstrated that there is a cooperative interaction between ligand occupancy of the *α*V*β*3 integrin and stimulation of intracellular signaling mechanisms by insulin-like growth factor I (IGF-I). This response occurs in both endothelial and vascular smooth muscle cells (VSMCs). We showed that hyperglycemia stimulated increases of *α*V*β*3 ligands and this resulted in increased *α*V*β*3 ligand occupancy and stimulation of tyrosine phosphorylation of *β*3 subunit. Inhibition of ligand occupancy of *α*V*β*3 inhibits pathophysiologic responses of endothelium [17] even in the presence of hyperglycemia [18]. Based on these findings this study was undertaken to test the hypothesis that inhibiting ligand occupancy of *α*V*β*3, using an antibody that binds specifically to the *β*3 subunit, might attenuate the early pathophysiologic changes that occur in diabetic nephropathy, particularly those that are related to endothelial dysfunction.

## 2. Methods

### 2.1. Materials

Polyvinyl difluoride membranes (Immobilon P) were purchased from Millipore Corporation (Billerica, MA). Autoradiographic film was obtained from Pierce (Rockford, IL). West Pico chemiluminescence reagent was purchased from Pierce (Rockford, IL). The monoclonal phosphotyrosine antibody (pY-99) was purchased from Santa Cruz Biotech (Santa Cruz, CA). The anti-TGF-*β* and collagen type IV antibodies were purchased from Abcam (Cambridge, MA). The anti-*α*V integrin antibody (AB1930) was purchased from Chemicon (Millipore, Billerica, MA). The anti-*β*3 polyclonal antibody used to immunoprecipitate or immunoblot for *β*3 (R2949) was prepared as described previously [18]. Gradient gels (4–14%) were purchased from Pierce (Rockford, IL). The anti-actin antibody was purchased from Cell Signaling Technology (Danvers, MA). Human dermal microvessel endothelial cells were obtained from Invitrogen (Life Technologies, Grand Island, NY). Attachment factor (S-006100), medium 131 (M131), microvessel growth supplement (MVGS; S-005), and trypsin (R-001) were also purchased from Invitrogen. Trypsin neutralization solution (TNS) was purchased from Clonetics (Lonza, Allendale, NJ).

All other reagents were purchased from Sigma Chemical Company (St. Louis, MO) unless otherwise stated.

### 2.2. Cell Culture

Human microvessel dermal endothelial cells (HMVECs) were grown in medium M131 with microvessel growth supplement and L-glutamine (10 mM) (M131-GM) on 10 cm dishes previously coated with endothelial cell-specific attachment factor (MVGS; Invitrogen). To coat the plates 5 mLs of the attachment factor was added to each plate and the plate was covered and then left for 30 minutes at 37°C. Excess attachment factor was removed and the plates were left to dry. Plates were sealed and stored at 4°C for up to one month. M131-GM was also supplemented with D-glucose to a final concentration of 20 mM where indicated. Cells were plated (at 200,000 cells/plate) and grown for 3 days in M131-GM; then, where indicated, the medium was supplemented with D-glucose to a final concentration of 20 mM for a further 3-4 days. Once confluent, cells were incubated overnight in M131 (with no MVGS) ± D-glucose (final 5 or 20 mM). The following day quiesced monolayers were treated with the anti-*β*3 (C-loop) antibody (see below) (1 *μ*g/mL) for 4 hours prior to lysis in a modified radio immunoprecipitation assay (RIPA) buffer. Equal amounts of protein lysates (200 *μ*g) were immunoprecipitated with a polyclonal anti-*β*3 antibody (R2949) and immune complexes were collected using protein A Sepharose and then separated by SDS-PAGE (8%). *β*3 phosphorylation was visualized following immunoblotting with an anti-phosphotyrosine antibody (pY-99). Equal amounts of protein lysate (containing 20 *μ*g protein solubilized in 45 *μ*L of Laemmli buffer) were also separated by SDS-PAGE (8%) and immunoblotted directly with the anti-*β*3 antibody. Western immunoblots were digitized and relative *β*3 phosphorylation was calculated using Image J and expressed as average arbitrary scanning units for each treatment.

### 2.3. Generation of the Mouse Monoclonal Anti-*β*3 (C-Loop) Antibody

The monoclonal antibody that binds to a specific epitope located between amino acids 177–183 of the human *β*3 integrin (termed the C-loop) was generated and purified as described by Maile et al. [19] except that after ammonium sulfate precipitation the material was passed directly over a protein G affinity column. Purity of the material was assessed by SDS-PAGE followed by silver staining and the concentration of antibody was assessed using an antigen-specific ELISA [8]. The purified mouse monoclonal antibody was diluted to 8.5 mg/mL in phosphate buffered saline, pH 7.0 and stored at −20°C. The purified antibody is referred to hereafter as the anti-*β*3 (C-loop) antibody.

### 2.4. Animals

Male Sprague Dawley rats (Charles River, Wilmington, MA) (*N* = 45) were housed (2 rats/cage) under 12-12-hour light-dark conditions, with free access to food and water. All protocols were approved by the University of North Carolina Chapel Hill Animal Care and Use Committee and adhere to the National Institutes of Health Guidelines. They were given access to food and water.

### 2.5. Diabetes Induction Protocol

All rats were fasted for 4 hours; then 15 were given a single intraperitoneal injection of vehicle (0.1 M sodium-citrate buffer, pH 4.5) and 30 were given streptozotocin (STZ; 50 mg/kg) in vehicle. Hyperglycemia was confirmed one week later using tail vein blood and a FreeStyle Lite glucose meter (Abbott Laboratories, Abbott Park, IL, USA) in the STZ-treated animals. At that point daily injections of insulin (Novolin N NPH, Novo Nordisk A/S, Bagsvaerd, Denmark) of 8 units/kg were commenced (intraperitoneal injection) and continued throughout the study. The rats were maintained in a diabetic state for 4 weeks before treatment was initiated. They were then assigned to one of 2 treatment groups. One group (*N* = 15) received saline and one (*N* = 15) received the anti-*β*3 (C-loop) antibody intraperitoneally 0.5 mg/kg every 72 hr for 8 weeks. Urine was collected after 4 and 8 weeks of treatment. Urine was collected by placing the rats in a clean cage covered with a fresh sheet of saran wrap. The rat was left in the cage until he urinated. Urine was collected using a sterile pipette tip and placed on ice.

Rats were weighed weekly throughout each study at the same time on each occasion.

### 2.6. Urinary Analysis

After collection urine samples were centrifuged at 13,000 rpm for 15 minutes. Supernatants were aliquoted and stored at −20°C prior to analysis. Rat urinary albumin, creatinine, type IV collagen, and nephrin were measured by specific ELISAs (Exocell, Philadelphia, PA). Assays were performed and data analyzed according to the manufacturer's instructions.

### 2.7. Assessment of Renal Biochemistry

After eight weeks of treatment the animals were anesthetized using Pentobarbital (Southern Anesthesia, West Columbia, SC; 80 mg/kg). Once under anesthesia the thorax was opened and the rat perfused with saline via an injection into the heart. The kidneys were then removed and dissected free of perinephritic fat and flash frozen in liquid nitrogen. At a later time point a section of kidney (approximately 1/4 of the total kidney containing cortex tissue only) was homogenized in RIPA buffer. The homogenates were centrifuged 13,000 ×g for 10 min to clear debris and the membrane proteins and cytosol (200 *μ*g/sample) were solubilized in 2x Laemmli buffer prior to separation by SDS-PAGE (TGF-*β* 4–12% gradient gel, thrombospondin-1 (TS-1) 6%, and collagen type IV 8%) followed by transfer to Immobilon P membranes. The membranes were incubated with antibodies for TGF-*β*, TS-1, and type IV collagen. Lysates from all of the animals in each group were analyzed. To determine if the antibody inhibited *β*3 phosphorylation, kidney homogenate (200 *μ*g) was immunoprecipitated with the anti-*β*3 antibody (R2949) using a 1 : 500 dilution. The proteins in the immunoprecipitate were separated by SDS-PAGE. After transfer to Immobilon P *β*3 phosphorylation was visualized by immunoblotting with anti-phosphotyrosine antibody (pY-99, Santa Cruz) at a 1 : 1000 dilution.

### 2.8. Anti-*β*3 (C-Loop) Antibody Binding to Rat *β*3

Anti-*β*3 (C-loop) antibody binding to rat *β*3 was measured using control rat kidney lysates (prepared as described above) and biotinylated form of the anti-*β*3 (C-loop) antibody. As a negative control lysates prepared from Chinese Hamster Ovary (CHO) cells that do not express *β*3 (CHO-K1) were purchased from ATCC and grown according to the accompanying instructions in alpha MEM plus 10% fetal bovine serum and penicillin and streptomycin. The CHO-K1 cells were grown to confluency in 10 cm dishes and incubated overnight in serum-free medium prior to lysis in RIPA. Clarified lysates were prepared by centrifuging at 13,000 rpm for 10 minutes at 4°C.

The anti-*β*3 (C-loop) antibody was biotinylated using EZ-link sulfo-NHS biotin labeling kit (Pierce) according to the manufacturer's instructions. Briefly, 1 mL of a 2 mg/mL solution of antibody was incubated with 27 *μ*L of a 10 mM biotin solution (designed to achieve a 20-fold molar excess of biotin to result in 4-5 biotin molecules/IgG molecule). After incubation the buffer was exchanged and free biotin was removed using the desalting column provided.

The rat lysates and CHO K-1 lysates (200 *μ*g/lysate) were immunoprecipitated with an anti-*α*V antibody (3 *μ*L/sample) and immune complexes captured using protein A beads were separated by SDS-PAGE (8%). Lysates were then immunoblotted with the biotinylated preparation of the anti-*β*3 (C-loop) antibody (1 : 1000 dilution) for 1 hour at room temperature. The binding of the biotinylated anti-*β*3 (C-loop) antibody was visualized following incubation with avidin-HRP (Jackson Immunoresearch, West Grove, PA) and then exposed to enhanced chemiluminescence reagent and autoradiographic film. To confirm that the band recognized by the biotin anti-*β*3 (C-loop) antibody was *β*3, lysates from the same preparations were separated directly by SDS-PAGE and then immunoblotted with a polyclonal anti-*β*3 antibody.

### 2.9. Measurement of Protein Concentration

Protein concentrations in all lysates were assessed using the bicinchoninic acid (BCA) kit from Pierce according to the manufacturer's instructions.

### 2.10. Quantification of Immunoblots

Western immunoblots were digitized by scanning on a flatbed scanner (HP Office Jet Pro 8600) and relative amounts of protein or *β*3 phosphorylation were calculated using Image J (Rasband, W.S., ImageJ, U.S. National Institutes of Health, Bethesda, MD, USA, http://imagej.nih.gov/ij/, 1997–2012) and expressed as average arbitrary scanning units for each treatment. To compare across multiple blots a standard loading control was loaded on each gel and bands on each gel were normalized to this control.

### 2.11. Statistical Analysis

Graphpad Prism (Graphpad Software, La Jolla, CA) was used for all statistical analyses. The statistical significance of the differences between treatment groups was compared using a two-tailed Student's *t*-test with *P* < 0.05 being considered significant.

## 3. Results

### 3.1. Characterization of Diabetic Rats

The average weight of all the rats in each group was not statistically different at the start of the study. At 8 or 12 weeks the nondiabetic control rats had gained significantly more weight than the diabetic rats (Table 1). There was no significant difference between the antibody- and vehicle-treated diabetic rats at the end of the study. The glucose levels of all the rats in each group were not significantly different at the start. One week after STZ treatment the glucose levels of the vehicle-treated diabetic rats and the rats to be treated with the anti-*β*3 (C-loop) antibody had increased significantly compared to control nondiabetic control rats, but there was no significant difference between the two groups of diabetic rats. At the end of the study the average glucose level in the control rats was not increased, but it remained elevated in the vehicle and anti-*β*3 (C-loop) antibody treated diabetic rats. There was no significant difference between the mean glucose values for the two diabetic groups at the end of the study (*P* = 0.28).

### 3.2. C-Loop Antibody Binds Rat to *β*3

**Figure 1:**
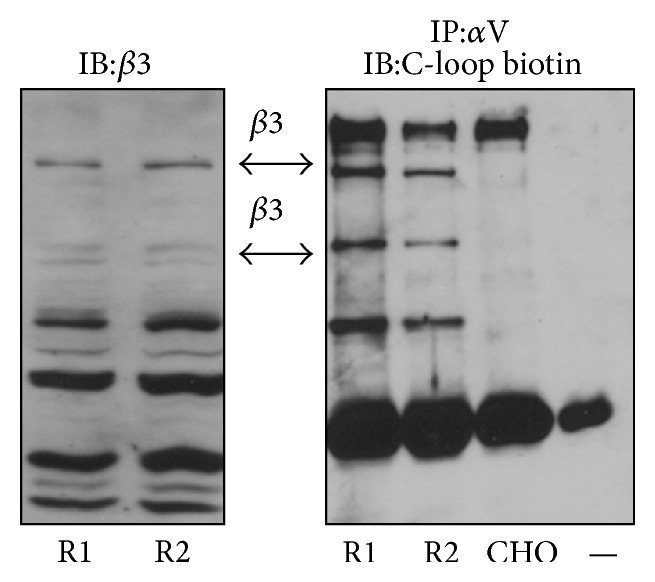
Biotin labeled C-loop antibody binding to *β*3 in rat kidney lysates from two diabetic rats (R1 and R2) as well as lysate from CHO cells (that do not express *β*3) was immunoprecipitated (IP) with an anti-*α*V antibody and then immunoblotted (IB) with a biotin labeled C-loop antibody (C-loop biotin). RIPA buffer alone was used as an additional negative control (−). Binding of the C-loop antibody to *β*3 in the kidney lysate but not to the CHO lysate was visualized with avidin HRP and enhanced chemiluminescence. To demonstrate that the specific band recognized by the C-loop biotin antibody corresponded to *β*3, lysates were also immunoblotted with an anti-*β*3 antibody.

Prior to testing the effect of the anti-*β*3 (C-loop) antibody in rats we wanted to demonstrate that it bound to rat *β*3. To demonstrate binding of the antibody specifically to rat *β*3 (associated with *α*V since this represents the active integrin unit), lysates from normoglycemic rat kidney were immunoprecipitated with an anti-*α*V antibody and then probed with a biotin-conjugated anti-*β*3 (C-loop) probe. CHO cells that express *α*V, but do not express *β*3, were used as a negative control [20]. In Figure 1 immunoblotting with the biotin labeled anti-*β*3 (C-loop) probe revealed several protein bands immunoprecipated with *α*V that were not apparent in the negative control lane. To confirm the identity of the bands detected with the biotinylated anti-*β*3 (C-loop) probe the rat kidney lysates were also probed with an anti-*β*3 antibody (right hand panel) and bands of a corresponding molecular weight were detected.

### 3.3. Urinary Albumin

**Figure 2:**
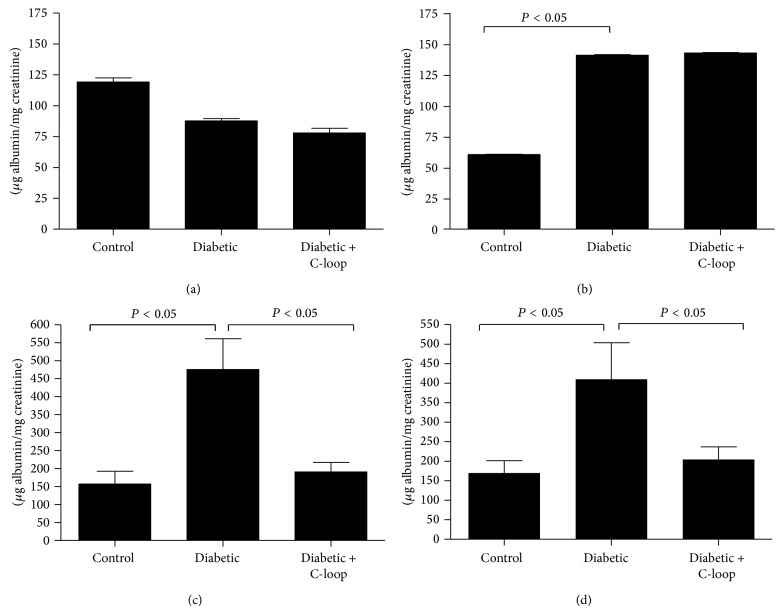
Urinary albumin and creatinine were measured in spot urine samples from all rats from each group (*n* = 15) at 4 time points (at the start of study week 0 (a), 4 weeks after the induction of hyperglycemia (b); control, 8 weeks of diabetes + with 4 wks vehicle (diabetic) or C-loop antibody treatment (c); control, 12 weeks of diabetes with 8 wks vehicle (diabetic); or 8 weeks of C-loop antibody treatment (d)). The results are shown as *μ*g albumin/mg creatine (mean ± SEM). *P* < 0.05 when the vehicle-treated hyperglycemic animals (diabetic) were compared with controls (con) or the hyperglycemic animals treated with the C-loop antibody (diabetic + C-loop).

The albumin to creatinine ratios (UACR) were similar in all 3 groups of rats at baseline (Figure 2(a)). After 4 weeks of hyperglycemia without treatment both groups of diabetic rats had major increases in UACR (Figure 2(b)). During the subsequent 8 weeks the vehicle-treated rats increased from 161 ± 24 to 457 ± 85 *μ*g/mg creatinine whereas the rats treated with the anti-C-loop antibody had levels of urinary albumin that were not significantly different from the nondiabetic rats (190 ± 27 *μ*g/mg creatinine compared to 157 ± 27 *μ*g/mg creatinine) (Figure 2(c)). At the end of the study (12 weeks of hyperglycemia + 8 weeks of antibody treatment) urinary albumin levels were 410 ± 96 *μ*g/mg creatinine in the vehicle-treated diabetic rats compared to 170 ± 32 *μ*g/mg creatinine in the antibody-treated group and 204 ± 33 *μ*g/mg in the nondiabetic group (Figure 2(d)). The difference between the two diabetic groups is highly significant and indicates that the antibody inhibits the increase in excretion of albumin that occurs between 4 and 12 weeks of diabetes.

### 3.4. Urinary Nephrin

**Figure 3:**
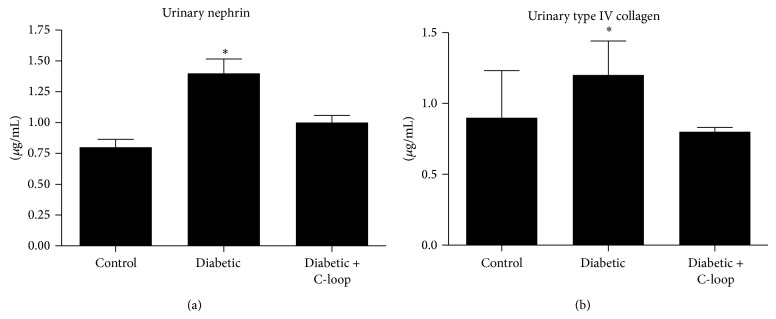
Urinary nephrin (a) and type IV collagen (b) were measured on spot urine samples obtained at the end of 12 weeks of treatment. The results are shown as *μ*g/mg creatinine (mean ± SEM). ^*^ *P* < 0.05 when the vehicle-treated diabetic animals are compared to the diabetic animals treated with the anti-C-loop antibody. ^*^ *P* < 0.05 when the control nondiabetic animals are compared to the vehicle-treated diabetic animals.

Nephrin was also measured in the urine as an index of podocyte damage. There was a substantial increase in nephrin excretion in the diabetic animals treated with vehicle compared to nondiabetic. In contrast the animals that received the anti-*α*V*β*3 antibody for the last 8 weeks of the study had no increase and the result was significantly less than vehicle-treated rats (Figure 3).

### 3.5. Urinary Type IV Collagen

To determine if the excretion of other proteins was altered urinary type IV collagen was measured. It was within the normal range in the nondiabetic animals and it increased 1.9-fold in the diabetic animals treated with vehicle after 12 weeks whereas the mean ± SE value in the animals that received the anti-*β*3 antibody was significantly less (*P* < 0.01) and not different when compared to the nondiabetic animals (Figure 3).

### 3.6. Inhibition of Profibrotic Changes Induced by Hyperglycemia in Kidney Lysates

**Figure 4:**
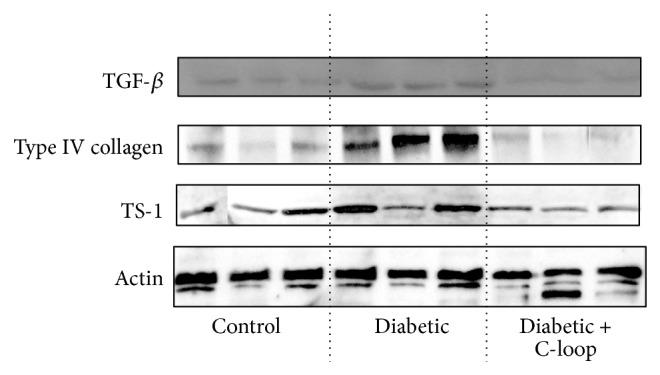
Biochemical analysis of kidney lysates. Kidney lysates from several rats in each group (*n* = 8-9) were prepared and equal amounts of total protein were separated by SDS-PAGE prior to immunoblotting (IB) with either an anti-TGF-*β*, anti-type IV collagen, or anti-TS-1 antibody. To demonstrate that any difference in protein levels was not due to a difference in the total amount of lysate, these extracts were also immunoblotted with an anti-actin antibody. The results from representative lysates of each group are shown. For each treatment group the protein band intensities from all the rats in the group were quantified using scanning densitometry. The arbitrary scanning units (mean ± SEM) are shown in Table 2.

Immunoblotting of kidney lysates revealed that TGF-*β*, TS-1, and type IV collagen were increased significantly in response to 12 weeks of diabetes in the animals that received vehicle compared to nondiabetic control animals (Table 2 and Figure 4). In contrast, the animals that received the active antibody showed no statistically significant changes compared to nondiabetic, control animals and their values were significantly less than the animals that received vehicle (Table 2 and Figure 4).

### 3.7. Demonstration of Inhibition of Target Activation

**Figure 5:**
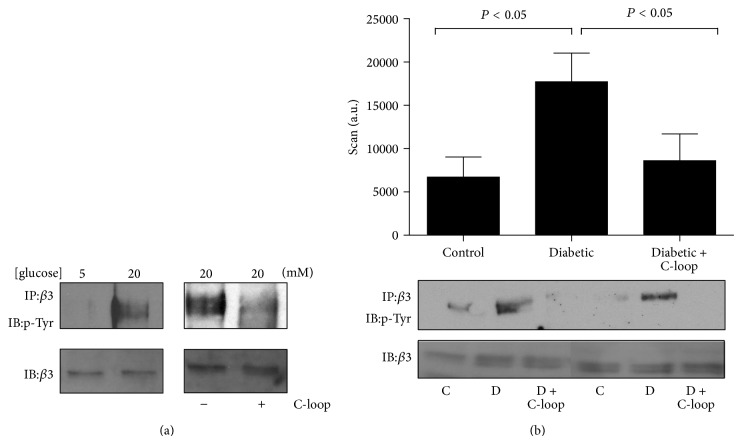
(a) Hyperglycemia increases *β*3 phosphorylation in microvascular endothelial cells. Left panel: lysates from microvascular endothelial cells grown in 5 or 20 mM glucose were immunoprecipitated (IP) with an anti-*β*3 antibody and immunoblotted (IB) with an anti-phosphotyrosine antibody (p-Tyr). To demonstrate that equal amounts of *β*3 in each treatment condition were analyzed, equal amounts of protein from the two lysates were immunoblotted directly for *β*3. Right panel: microvascular endothelial cells grown in 20 mM glucose were incubated with (+) or without (−) the C-loop *β*3 antibody for 4 hours prior to being immunoprecipitated (IP) with an anti-*β*3 antibody and immunoblotted (IB) with an anti-phosphotyrosine antibody (p-Tyr). To demonstrate equal amounts of *β*3 in each treatment condition, equal amounts of protein from the two lysates were immunoblotted directly for *β*3. (b) *β*3 phosphorylation in rat kidney lysates. Kidney lysates from control rats, vehicle-treated diabetic rats, or diabetic rats treated with C-loop antibody were immunoprecipitated (IP) with an anti-*β*3 antibody, separated by SDS-PAGE, and immunoblotted (IB) with an anti-phosphotyrosine antibody (p-Tyr). To demonstrate that this was not due to differences in the total amount of *β*3 in each sample equal amounts of protein were also immunoblotted directly for total *β*3. For each treatment group the phosphorylated protein band intensities from all the rats in the group (*n* = 15) were quantified using scanning densitometry. The arbitrary scanning units (mean ± SEM) are represented graphically and an example from 2 animals from each treatment group is shown below the graph. *P* < 0.05 when the extent of *β*3 phosphorylation is compared between the control (con) and the untreated diabetic group (D), or the untreated diabetic group is compared to the diabetic group treated with C-loop antibody (D + C-loop).

Consistent with our previous results in other *α*V*β*3 expressing vascular cell types (e.g., SMCs), exposure of HMECs to 20 mM glucose resulted in higher (3.1 ± 0.2-fold; *P* < 0.05) levels of detectable *β*3 phosphorylation compared with cells incubated in 5 mM glucose (Figure 5). This increased phosphorylation could be inhibited (a 2.1 ± 0.11-fold decrease; *P* < 0.05) by incubating the cells with the anti-*β*3 (C-loop) antibody (Figure 5(a)).

The kidneys were obtained from the animals at sacrifice as described in methods. As shown in Figure 5(b), the *β*3 phosphorylation band intensity was increased in the kidney lysates obtained from diabetic compared to nondiabetic control animals (a 2.6-fold increase). To demonstrate target engagement and the biologic activity of the anti-*β*3 (C-loop), its ability to inhibit *β*3 phosphorylation in intact animals was determined. Treatment of the rats with the anti-C-loop antibody reduced the hyperglycemia-induced increase in *β*3 phosphorylation to a level that was similar to that in the control animals (Figure 5(b)).

## 4. Discussion

These findings demonstrate that inhibiting *α*V*β*3 integrin activation with a specific monoclonal antibody that is directed against its C-loop domain, as measured by inhibition of *β*3 phosphorylation, inhibited the progression of proteinuria in rats with STZ-induced diabetes mellitus. Importantly, the antibody inhibited a further increase proteinuria in rats that had been diabetic for 4 weeks and allowed them to maintain levels that were similar to nondiabetic control animals. This finding was confirmed by demonstrating that the increase in urinary collagen and nephrin that occurs with diabetes was also prevented. The results also show that hyperglycemia-induced increases in the expression of TGF-beta and TS-1, which have been linked to specific pathophysiologic mechanisms that are activated in diabetic nephropathy, were also inhibited [3, 21–23]. Type IV collagen, a protein known to be an index of ongoing kidney damage, [13, 24–27], and TGF-*β*, a protein that increases with mesangial expansion [28], were also significantly altered in rats administered the anti-*α*V*β*3 antibody. This suggests that stimulation of ligand occupancy of the *α*V*β*3 integrin is involved in the pathophysiologic changes that occur during the early stages of the development of diabetic nephropathy and these changes which indicate the loss of normal barrier function can be prevented from progressing by targeting this integrin.

Exposure of endothelial cells to hyperglycemia results in increases of the extracellular concentrations of vitronectin [17], osteopontin [29], and TS-1 [30]: all of which are *α*V*β*3 ligands. These increases result in increased binding to *α*V*β*3 and activation of tyrosine phosphorylation of *β*3 cytoplasmic domain leading to recruitment of signaling molecules to these phosphotyrosines [31]. In this study we demonstrate that our antibody inhibited the *β*3 subunit tyrosine phosphorylation indicating that it inhibited ligand-induced activation of *α*V*β*3. In vascular endothelial cells increased activation of the *α*V*β*3 integrin has been shown to lead to enhancement of the ability of IGF-I to stimulate cell proliferation and migration. Additionally, it was shown to enhance IGF-I stimulated p52Shc and MAP kinase phosphorylation [17]. More relevant to these studies administration of a monoclonal antibody that disrupted IGF-I signaling system in hyperglycemic rats was effective in inhibiting the increase in capillary permeability [32]. This was shown by demonstrating its ability to inhibit Evans blue dye leakage into retina after four weeks of treatment. Therefore it appears that inhibiting IGF-I stimulated signaling is effective in inhibiting altered capillary permeability that occurs as a function of hyperglycemia. To the extent that endothelial dysfunction contributes to the loss of renal barrier function we conclude that this antibody may be effective in inhibiting the alterations in permeability that occur in the endothelial component of the basement membrane by inhibiting the interaction between *α*V*β*3 and IGF-I.

More recently another manifestation of endothelial cell dysfunction, namely, abnormal angiogenesis, has also implicated the progression of diabetic nephropathy [33]. Inhibitors of angiogenesis inhibit the progression of DN in rat and mouse models. *α*V*β*3 is overexpressed in activated endothelial cells and inhibitors of *α*V*β*3 have been shown to prevent angiogenesis [34–39]. In particular, the highly selective *α*V*β*3 RGD antagonist RGDechHCit has been shown to inhibit angiogenesis in rat and mice models [40]. Thus it is possible that blocking *α*V*β*3 activation with the C-loop antibody reduced the progression of proteinuria by preventing abnormal angiogenesis.

Earlier studies have shown that *α*V*β*3 integrin expression is increased in epithelial and mesangial cells derived from diabetic kidneys [10, 13]. Some studies have also reported podocyte *α*V*β*3 expression [14]. The *α*V*β*3 receptor antagonist tumstatin was shown to inhibit glomerular hypertrophy and mesangial expansion in the early stages of diabetic nephropathy [34]. However its effects may not be specific for *α*V*β*3 since tumstatin can bind to other integrins such as *α*V*β*1. Blocking *α*V*β*3 activation in podocytes by indirectly inhibiting the secretion of soluble urokinase-type plasminogen activator receptor reduced lipopolysaccharide induced proteinuria, suggesting that this receptor may be involved in the loss of podocyte barrier function [41]. Whether *α*V*β*3 is stimulated in response to hyperglycemia-induced increases in ligand occupancy [18] in this cell type and whether the same type of signaling mechanism that has been shown to be activated in endothelial cells by hyperglycemia and IGF-I [17] is present in these cell types are unknown. Our results demonstrating a reduction in urinary nephrin suggest that the anti-*β*3 antibody is inhibiting the effect of hyperglycemia on podocyte function, but to determine the extent to which inhibiting ligand occupancy of podocyte *α*V*β*3 is participating this process will require further studies.

One limitation in the development of therapeutics for the treatment of diabetic nephropathy is the lack of good animal models that accurately develop progressive renal disease similar to that seen in humans (i.e., both increases in albuminuria and a progression to decreased GFR) in the context of both type 1 and type 2 diabetes. In this study we have used a well-characterized model of streptozotocin (STZ) induction of hyperglycemia to mimic parameters of type 1 diabetes. However, as reported by others, these animals do not progress beyond the early stages of disease with respect to renal function; they display the proteinuria characteristic of early human diabetic kidney disease but do not display decreased GFR or other impairments characteristic of advanced disease [42]. While our study provided evidence that targeting *α*V*β*3 prevented and reversed proteinuria associated with hyperglycemia, lack of histological and ultrastructural studies limits the ability to interpret how to target the *α*V*β*3 integrin functions at the cellular or molecular level to prevent or reverse this clinical end-point. However, we have shown recently in a different animal model of type 2 diabetes, hyperglycemic hypercholesteremic pigs studied over 20 weeks of diabetes [43], that targeting the *α*V*β*3 integrin, using the same antibody as used in this study, was associated with a reduction in several histological changes associated with diabetic nephropathy including mesangial matrix expansion, glomerular basement membrane thickening, and increases in podocyte foot width. Furthermore, as in this study, these histological changes were also associated with the prevention of the development of proteinuria. While these two models are quite distinct taken together, the data suggest that inhibiting the activation of *α*V*β*3 is associated with the attenuation of changes in kidney structure and function associated with diabetes.

A further limitation of this study is the effect of the antibody on blood pressure. Given the importance of hypertension in determining glomerulosclerosis and proteinuria [44] it is possible that targeting *α*V*β*3 resulted in changes in blood pressure that indirectly resulted in the differences in proteinuria between the groups of animals.

In summary, blockade of the *α*V*β*3 integrin in diabetic rat kidney results in reversal of proteinuria and inhibition of synthesis of several proteins that have been shown to be induced in diabetic nephropathy. The fact that these changes are reversible suggests that inhibition of *α*V*β*3 activation may have a role in the early stage of the disease by attenuating changes that lead to loss of glomerular function. Taken together with the results of our other recent study [43] these studies suggest that this antibody may have efficacy in preventing the progression of diabetic nephropathy.

## Figures and Tables

**Table 1:** Characteristics of study animals.

	Body weight (grams ± SEM)		Glucose (mg/dL ± SEM)		
	Start	12 wks diabetes/8 wks treatment	Start	1 wk diabetes	12 wks diabetes/8 wks treatment
Control nondiabetic	273 ± 33	589 ± 15	107 ± 3	108 ± 5	137 ± 23
Diabetic vehicle-treated	273 ± 20	446 ± 18	115 ± 6	352 ± 36	375 ± 36
Diabetic antibody-treated	270 ± 21	425 ± 23	113 ± 7	414 ± 25	425 ± 28

*N* = 15 for each group.

**Table 2:** Analysis of fibrotic proteins in kidney lysates.

	Control	Fold increase between control and diabetic	Diabetic	Fold difference between diabetic and diabetic + C-loop	Diabetic + C-loop
TGF-*β*	7722 ± 1280 *N* = 8	2.3	17564 ± 2122∗ *N* = 8	2.8	6242 ± 641.6^+^ *N* = 8
Collagen type IV	4892 ± 1457 *N* = 8	3.7	18249 ± 1165∗ *N* = 9	2.3	7810 ± 1849^+^ *N* = 8
TS-1	6261 ± 1205 *N* = 8	2.8	17735 ± 3795∗ *N* = 9	2.2	8032 ± 1051^+^ *N* = 9

Mean (±SEM) of arbitrary scanning units (obtained from analysis of western immunoblots) and fold difference between treatment groups. *N* for each analysis is indicated in the table. ∗*P* < 0.05 when levels in control nondiabetic animals are compared with diabetic animals that received vehicle and when the levels from those animals are compared with the levels obtained from diabetic rats treated with the C-loop antibody. ^+^ *P*: NS when diabetic + C-loop antibody treatment is compared to control animals.
